# An NF-κB- and Therapy-Related Regulatory Network in Glioma: A Potential Mechanism of Action for Natural Antiglioma Agents

**DOI:** 10.3390/biomedicines10050935

**Published:** 2022-04-19

**Authors:** Evrysthenis Vartholomatos, Stefania Mantziou, George A. Alexiou, Diamanto Lazari, Chrissa Sioka, Athanassios Kyritsis, Georgios S. Markopoulos

**Affiliations:** 1 Neurosurgical Institute, Faculty of Medicine, School of Health Sciences, University of Ioannina, 451 10 Ioannina, Greece; eyrys.varth@gmail.com (E.V.); alexiougr@gmail.com (G.A.A.); csioka@uoi.gr (C.S.); thkyrits@uoi.gr (A.K.); 2 Haematology Laboratory, Unit of Molecular Biology, University Hospital of Ioannina, 455 00 Ioannina, Greece; stefanimantz@gmail.com; 3 Department of Neurosurgery, University of Ioannina, 451 10 Ioannina, Greece; 4 Laboratory of Pharmacognosy, Faculty of Health Sciences, School of Pharmacy, Aristotle University of Thessaloniki, 541 24 Thessaloniki, Greece; dlazari@pharm.auth.gr; 5 Clinical Laboratory of Nuclear Medicine, University of Ioannina, 451 10 Ioannina, Greece

**Keywords:** glioma, anticancer therapy, NF-κB, natural antiglioma agents, CD markers

## Abstract

High-grade gliomas are among the most aggressive malignancies, with significantly low median survival. Recent experimental research in the field has highlighted the importance of natural substances as possible antiglioma agents, also known for their antioxidant and anti-inflammatory action. We have previously shown that natural substances target several surface cluster of differentiation (CD) markers in glioma cells, as part of their mechanism of action. We analyzed the genome-wide NF-κB binding sites residing in consensus regulatory elements, based on ENCODE data. We found that NF-κB binding sites reside adjacent to the promoter regions of genes encoding CD markers targeted by antiglioma agents (namely, CD15/FUT4, CD28, CD44, CD58, CD61/SELL, CD71/TFRC, and CD122/IL2RB). Network and pathway analysis revealed that the markers are associated with a core network of genes that, altogether, participate in processes that associate tumorigenesis with inflammation and immune evasion. Our results reveal a core regulatory network that can be targeted in glioblastoma, with apparent implications in individuals that suffer from this devastating malignancy.

## 1. Introduction

Carcinogenesis is a step-by-step process through which normal cells acquire genetic and epigenetic alterations, and transform into malignant cells that form a tumor mass. Cancer is the second leading cause of human mortality worldwide, with almost 10 million deaths, or ~18% of total deaths, in 2020 [1]. Among them, central nervous system malignancies (CNSMs), including brain tumors (ICD codes C70–72), account for ~308,000 new cases and ~251,000 deaths, making it one of the deadliest types of cancer per case [2]. Central nervous system tumors have been historically classified based on their histological parameters, mainly as a result of the occurrence of malignancy from different brain tissues [3]. However, the latest classifications of CNSMs—the 2016 and 2021 updates from the World Health Organization—consider more advanced molecular characteristics that are now available in the post-genomic era, providing a more comprehensive catalog with usefulness in clinical management and treatment [2,4].

Glioblastoma (GBM) represents the most common malignant brain tumor. Despite intensive clinical investigation and the discovery of several novel treatments, patients’ median survival remains poor, in the range of 15 months [5]. The standard therapeutic approach involves surgical resection of the tumor mass, which is followed by radiotherapy with concurrent and adjuvant chemotherapy [6]. Temozolomide (TMZ) represents the standard primary chemotherapy for GBM [7]. The genetic heterogeneity that leads to diverse molecular mechanisms of tumor development makes it difficult to successfully treat GBMs. Cells that survive the first line of treatment eventually develop resistance, leading to recurring tumors that are resistant to both radiotherapy and chemotherapy [8].

In previous reports by our team, the natural substances moschamine [9], n-p-coumaroyl-serotonin [10], and deglucohellebrin [11] have been shown to exhibit significant antiglioma activity in vitro and low cytotoxicity in normal tissues in vivo, as shown in a zebrafish embryo toxicity model. An interesting feature of these natural substances is that they represent distinct paradigms of compounds with potent antioxidant and anti-inflammatory activities [12,13].

Nuclear factor kappa B (NF-κB) represents a family of transcription factors that play major roles in molecular circuits that associate carcinogenesis with inflammatory response [14]. It has been established that NF-κB plays a major role in glioma pathogenesis and chemoresistance [14]. Based on the above roles, NF-κB is a potent molecular target in glioblastoma [15].

In the present article, we report a molecular network that includes common targets of natural compounds and the NF-κB family of transcription factors. Our results suggest a possible common mechanism of action for natural compounds and a core regulatory network that may be further targeted in glioblastoma.

## 2. Materials and Methods

### 2.1. Sequence Analysis and Annotation

Transcription factor binding sites for the RELB subunit of the NF-κB family were extracted from ChIP-seq data available from the ENCODE (Encyclopedia Of DNA Elements) Project Consortium, and were further analyzed using bioinformatic tools available in the UCSC Genome Browser (http://genome.ucsc.edu/) [16,17,18], as well as the UCSC Table Browser [19]. Our data retrieval workflow included the following steps: First, ChIP-seq peaks that were generated as part of the ENCODE Project [20], were retrieved using the UCSC Table Browser (filtering the data available in the table wgEncodeRegTfbsClusteredV3) [21,22,23]. The presence of candidate cis-regulatory elements by ENCODE [24] was determined using the UCSC Table Browser, in the extracted ChIP-seq peaks. ChIP-seq peaks that reside in CREs were annotated in relation to transcription start sites (TSSs) using the Genomic Regions Enrichment of Annotations Tool (GREAT) (great.stanford.edu) [25]. The resulting data were further inspected and analyzed using the UCSC Genome Browser. The servers were last accessed on 2 March 2022.

### 2.2. Network Analysis

The regulatory network that includes common targets of natural compounds and NF-κB was constructed with the GENEMANIA prediction server tool (www.genemania.org), using the default settings that result in the generation of a number of 20 nearest-neighbor genes in the final network [26]. The involvement of the final gene list from the aforementioned network in cellular pathways was calculated by enrichment analysis in the Reactome pathway knowledgebase (www.reactome.org) [27]. Analysis was performed against Reactome version 79. In addition, the resulting gene set was analyzed for enrichment of Gene Ontologies, cellular pathways, and disease, using the Enrichr tool (https://amp.pharm.mssm.edu/Enrichr/) [28,29]. The servers were last accessed on 2 March 2022.

## 3. Results

### 3.1. The Binding Landscape of NF-κB in Consensus Regulatory Elements and in the Vicinity of Transcription Start Sites

Our initial goal was to establish the genome-wide binding landscape of NF-κΒ in regulatory regions. First, the ChIP-seq-related peaks for RELB were extracted from available ENCODE project consortium data. A total of 35,961 ChIP-seq peaks were further filtered for their presence in candidate cis-regulatory elements (cCREs) genomic regions. We found that 31,477 RELB binding sites (87.5% of the total binding sites) reside in cCREs (Table S1), and may have the potential to take part in the regulation of gene expression. To create an NF-κB binding landscape in the vicinity of genes, we performed GREAT analysis, finding the single nearest gene in the vicinity of each ChIP-seq peak, no more than a maximum extension of 5 Kb to the transcription start site (TSS). Several peaks (10,681 peaks, or 34% of total peaks) were found in the vicinity of 7927 genes (Figure S1 and Table S2, Supplementary Materials).

### 3.2. Natural Substances Target Cluster of Differentiation (CD) Markers That Contain NF-κB Binding Elements

**Figure 1:**
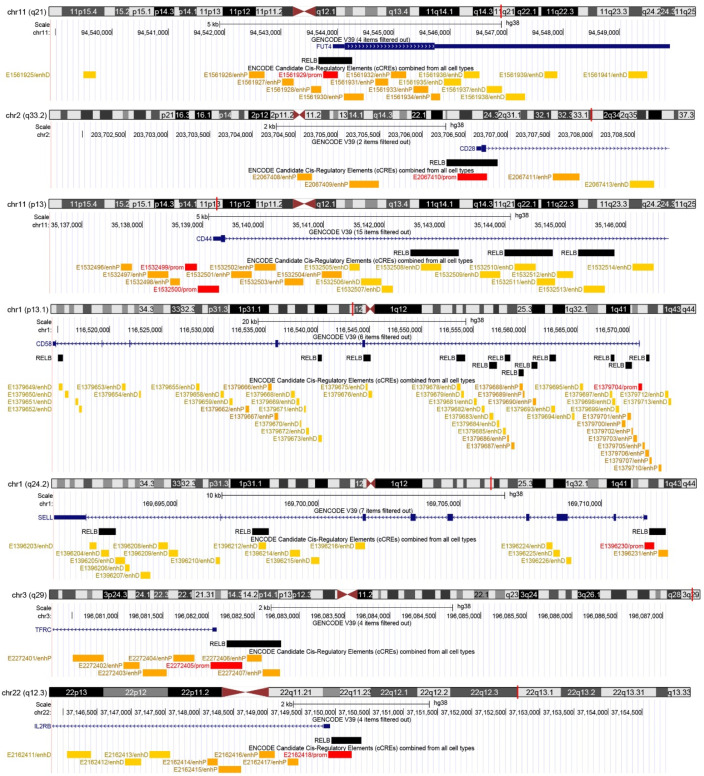
Binding of the NF-κB subunit RELB in glioma-therapy-associated CD markers. For every CD marker, a chromosome ideogram with chromosomal band location is shown as a vertical red line. Below each chromosome ideogram, parallel lines depict a chromosomal scale, the exact location of each gene transcriptional start site (based on GENCODE V39), and the positions of respective RELB binding sites and ENCODE candidate cis-regulatory elements.

Based on our previous results, we found several cluster of differentiation (CD) markers that are targeted by the natural compounds moschamine, n-p-coumaroyl-serotonin, and deglucohellebrin. Importantly, their expression has also been associated with the significant antiglioma activity of the compounds [9,10,11]. Since a known action of these compounds is their potent anti-inflammatory activity [12,13], a candidate common antiglioma mechanism may include the action of NF-κB—a potent molecular target in glioblastoma [15]. To this end, we screened the CD molecules targeted by moschamine (MM), n-p-coumaroyl-serotonin (CS), and deglucohellebrin (DGH) for the presence of NF-κB binding sites in cCREs. Most CD molecules targeted by CS, MM, and DGH—namely, CD15/FUT4, CD28, CD44, CD58, CD61/SELL, CD71/TFRC, and CD122/IL2RB—are also targets of NF-κB, at a distance to TSS of less than 5 Kb (Figure 1).

Notably, following manual verification in the UCSC Genome Browser, we found that the NF-κB peaks were either adjacent to TSSs (in CD15/FUT4, CD28, CD71/TFRC, and CD122/IL2RB), or the cis-regulatory region contained several NF-κB binding sites (3 in CD44, 12 in CD58, and 3 in CD61/SELL). As regards the regulatory potential of the NF-κB binding sites, six out of seven promoter-like cCREs (in CD15 CD28, CD58, CD61, CD71, and CD122) were associated with at least one NF-κB peak, while in CD44, NF-κB peaks were present in distal enhancer cCREs. Collectively, the action of the natural compounds CS, MM, and DGH is associated with altered expression of CD molecules that contain NF-κB binding sites in their cis-regulatory elements.

### 3.3. An Expanded Glioma-Therapy-Associated Network of CD Markers Containing NF-κB cCREs

**Figure 2:**
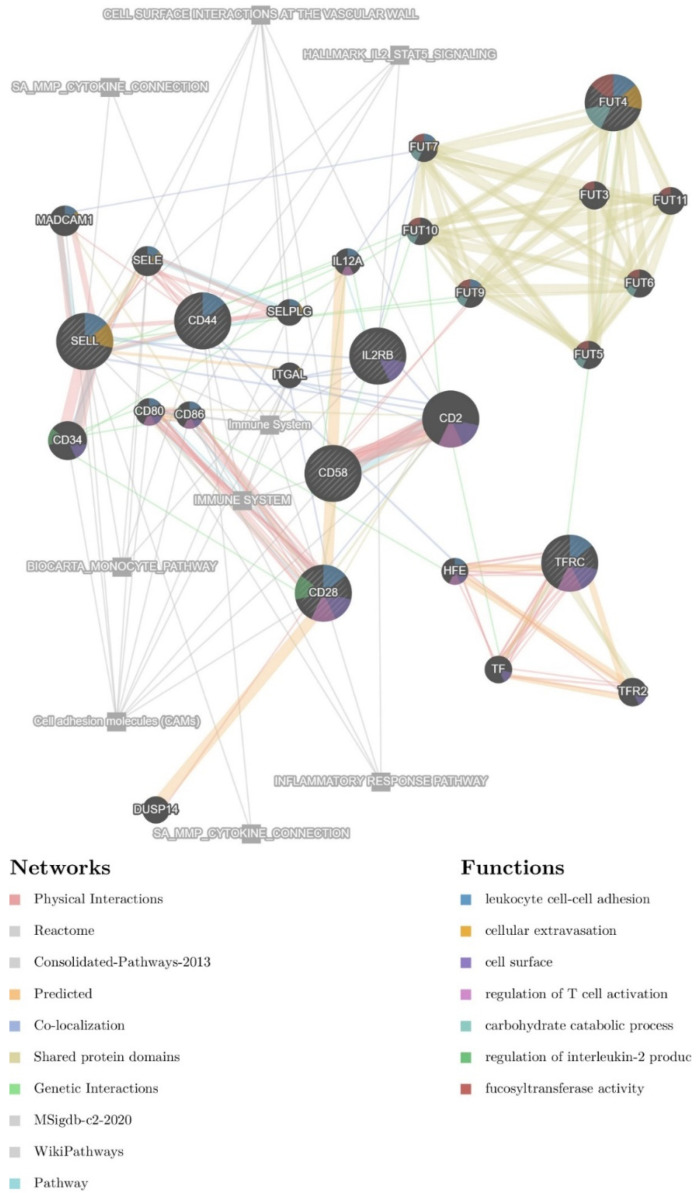
Interaction network containing surface markers that are targeted by NF-κB. The nodes indicate interaction. The color of each node designates a different type of interaction, explained in the bottom-left corner legend. Associated functions are designated with a colored pie chart within the respective circles representing individual proteins, explained in the bottom-right corner legend.

Next, therapy and NF-κB associated CD markers were used as an initial seed for the construction of a regulatory network also containing 20 resultant genes in the GeneMANIA web server. The potential glioma-therapy-related network (GTN) contains 27 genes (Figure 2). Associated pathways are also present in the final network. The resultant genes contain members of the FUT family (including FUT3, FUT5, FUT6, FUT7, FUT9, FUT10, and FUT11), other CD markers (e.g., CD2, CD34, CD80, CD86), and members of the selectin family (e.g., SELE, SELPLG), as well as TF, TFR2, HFE, DUSP14, ITGAL, IL12A, and MADCAM1.

### 3.4. Enriched Pathways Containing GTN Genes

**Figure 3:**
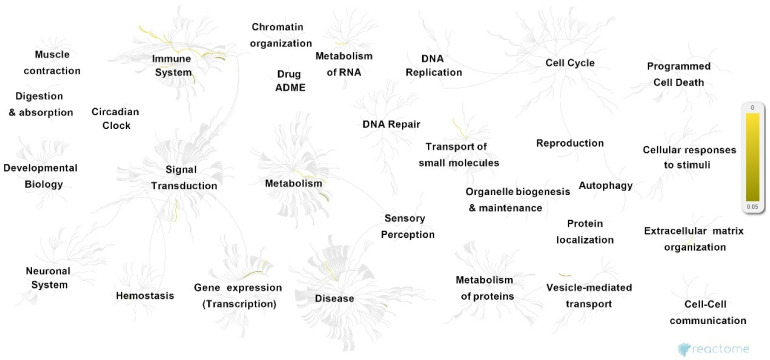
Overview of pathways associated with GTN genes, following Reactome analysis. Significantly enriched pathways (*p* < 0.05) are highlighted in yellow.

GTN genes were further characterized using two types of enrichment analysis. First, Reactome analysis was performed to provide an overview of the significant pathways and their associations (Figure 3 and Table S2, Supplementary Materials). The analysis revealed the 25 most enriched pathways (summarized in Table 1), most of which can be categorized in terms of highly associated cellular functions.

These pathways mostly take part in CD28- and PI3K/AKT-associated signaling (stimulation by the CD28 family CD28 co-stimulation; CD28-dependent Vav1 pathway CTLA4 inhibitory signaling; CD28-dependent PI3K/Akt signaling; constitutive signaling by aberrant PI3K in cancer; PI3K/AKT signaling in the cancer adaptive immune system; PI5P, PP2A, and IER3 regulate PI3K/AKT signaling; negative regulation of the PI3K/AKT network), immune response (immune system; RUNX3 regulates immune response and cell migration), iron homeostasis (iron uptake and transport; transferrin endocytosis and recycling), and inflammation-associated interleukin and cytokine signaling (interleukin-10 signaling; interleukin-35 signaling hemostasis; signaling by interleukin–integrin cell surface interactions; cytokine signaling in the immune system). In addition, several other independent pathways are also enriched, including blood group systems’ biosynthesis, insulin-like growth factor-2 mRNA binding proteins (e.g., IGF2BPs/IMPs/VICKZs) to bind RNA, and the metabolism of carbohydrates and cell surface interactions at the vascular wall.

**Figure 4:**
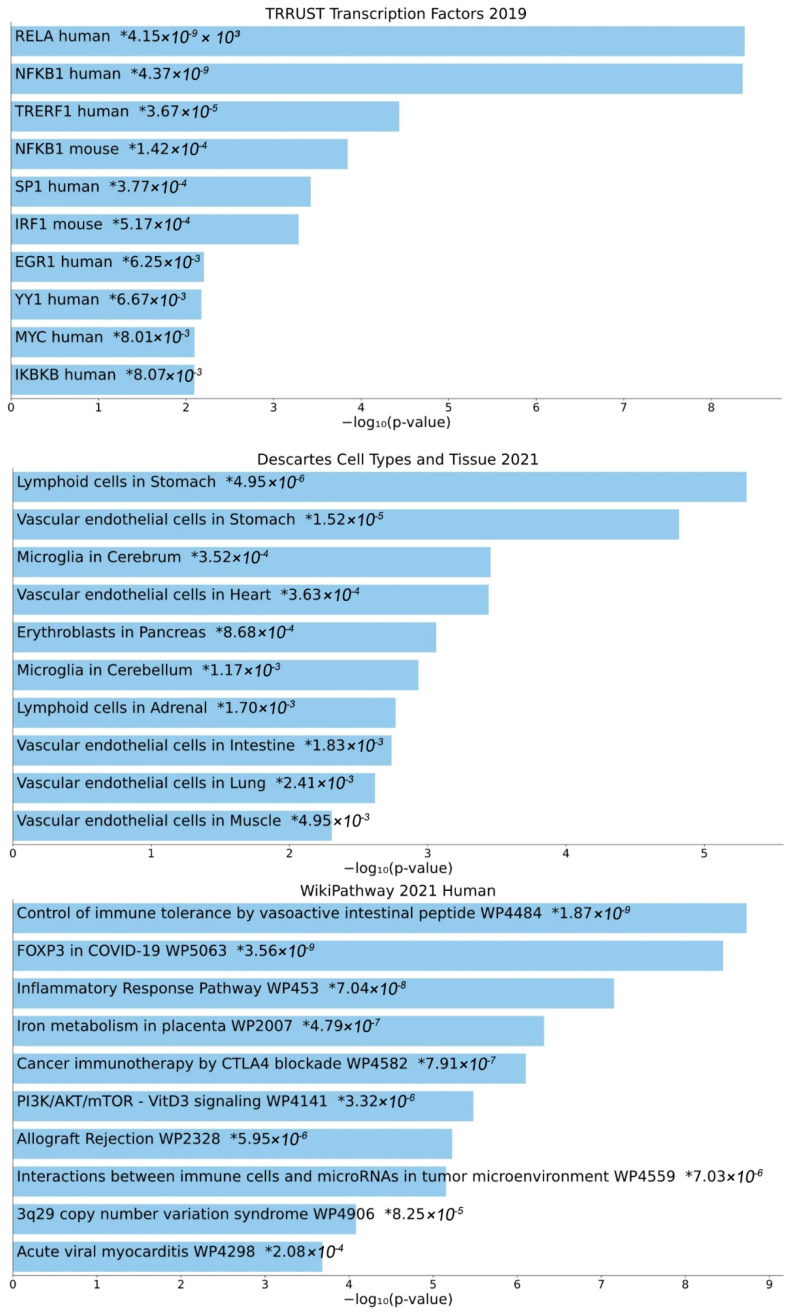
Transcription factors, cell types, and pathways associated with GTN genes. Top 10 enriched transcription factors (TRRUST Transcription Factor Database version 2019), cell types (Descartes Cell Types and Tissue Database version 2021), and pathways (WikiPathways Database version 2021), based on Enrichr analysis. Individual asterisks denote *p*-values.

Second, Enrichr analysis was performed for the enrichment of GTN genes in gene sets present in specific databases (Figure 4). The top 10 enriched transcription factors in TRRUST Transcription Factor Database version 2019 include several members of the NF-κB family (human RELA, human NFKB1, murine NFKB1, and human IKBKB). Importantly, microglia in the cerebrum and cerebellum are included among the tissues types in which GTN genes are expressed, from the Descartes Cell Types and Tissue Database version 2021. Enriched pathways, from the WikiPathways Database version 2021, among others, include cancer-related pathways (cancer immunotherapy by CTLA4 blockade WP4582; interactions between immune cells and microRNAs in the tumor microenvironment WP4559), immune-system-related pathways (control of immune tolerance by vasoactive intestinal peptide WP4484; allograft rejection WP2328), virus-related pathways (FOXP3 in COVID-19 WP5063; acute viral myocarditis WP4298), and inflammatory response pathway WP453, iron metabolism in placenta WP2007, PI3K/AKT/mTOR-VitD3 signaling WP4141, and 3q29 copy number variation syndrome WP4906.

## 4. Discussion

In the present report, we established the existence of a novel molecular network of common targets between natural compounds and the NF-κB transcription factor family. Our approach suggests that a possible common mechanism of action for natural compounds might involve the action of NF-κB, leading to a core regulatory network that acts in glioblastoma and is associated with chemosensitivity. The significance of our results is based on three lines of evidence:

First, the antiglioma action of the natural substances MM, CS, and DGH has been previously established [9,10,11]. The GTN gene set established in the present report is relevant to our previous findings, since they are expressed in microglia in the cerebrum and cerebellum (from Descartes Cell Types and Tissue 2021)—the tissue types that include most cells that transform into glioma. In addition, GTN genes are also associated with cancer-related immunotherapy (WP4582) and the interactions between immune cells and microRNAs in the tumor microenvironment (WP4559). The GTN gene set includes the majority of surface markers that we found to be altered following chemosensitivity of glioma cells, possibly following the potent antioxidant and anti-inflammatory activities of MM, CS, and DGH [12,13]. A possible limitation of our study is that while MM and CS share a highly similar structure, DGH is a bufadienolide with a different structure. On the other hand, the potential of substances with different structures to instigate similar biological outcomes might constitute an interesting finding, and may also suggest that other potent antioxidant and anti-inflammatory substances may have antiglioma activity, by affecting our suggested network.

Second, NF-κB promotes the survival and chemoresistance of glioblastoma, and there is evidence that this action may potentially be inhibited by several natural compounds. The transcription factor NF-κΒ plays an important role in (a) the survival and regulation of the cell cycle in glioblastoma [30], (b) resistance to chemotherapy [31,32,33], (c) the induction of cancerous stem cells [34] and aggressive phenotypes [35,36], and (d) invasion, angiogenesis [37] and metastasis of glioblastoma cells [38,39]. Inhibition of NF-κΒ is associated with increased sensitivity to chemotherapy [33], and has therefore been proposed as a strategy for treating glioblastoma [15]. Given that CS is known to inhibit NF-κB [40], and based on the common structural characteristics and the similarity in in vitro action [41,42], NF-κB is a potent candidate for glioblastoma treatment. Several members of the NF-κB family (i.e., human RELA, human NFKB1, murine NFKB1, and human IKBKB, based on the TRRUST Database) are included among the top 10 enriched TFs in GTN genes. In addition, the immune response, immune-system-related pathways (WP4484; WP2328), and inflammation-associated interleukin- and cytokine signaling (WP453) are among the pathways most associated with GTN genes, in both Reactome and Enrichr. NF-κΒ is known to play major roles in both immunity and inflammation [43], as well as in the interplay between inflammation and cancer [14]. In addition, NF-κB may also be associated with virus-related pathways, such as FOXP3 in COVID-19 (WP50630). There is a known interplay between FOXP3 and NF-κB, whereby FOXP3 can modulate NF-κB activity, and vice versa, leading to diverse physiological and pathological effects [44,45]. Both factors have been associated with severe COVID-19, while NF-κΒ targeting has been proposed as a treatment that might also involve some of the GTN genes [46,47]. Importantly, acute viral myocarditis (WP4298)—an additional enriched pathway with GTN genes—is associated with NF-κB-induced inflammation, in a regulatory network that also involves the microRNAs miR148a and miR-155 [48].

Third, MM, CS, and DGH action and NF-κB binding converge in GTN genes. To the best of our knowledge, this is the first study to examine whether the aforementioned CD markers contain NF-κB binding sites. The implications of such binding are the functional targeting by NF-κB and the potential to create feedback loops taking part in regulatory networks. FUT4/CD15 have been shown to be targeted by NF-κB—an observation that strengthens our arguments [49,50]. A drawback of the suggested mechanism is that it needs further experimental validation. We believe that additional studies would confirm the regulatory role of NF-κB in GTN genes.

Among the GTN genes, the most abundant are the members of the fucosyltransferase (FUT) family, including FUT4/CD15, FUT5, FUT6, FUT7, FUT9, FUT10, and FUT11. CD15 is a cancer-associated marker in brain malignancies [51], which is also present in normal [52] and cancer stem cells [53,54]. FUT5 and FUT6 have been associated with a PI3K-mediated aggressive phenotype in colorectal cancer [55]. FUT7 is induced by L-selectin to facilitate hematogenous carcinoma metastasis, which is supported by aggregated platelets and white blood cells [56], while in bladder urothelial carcinoma it is associated with epithelial–mesenchymal transition [57]. FUT9 has been associated with colon cancer progression both by induction of cell dedifferentiation into a stem-like state [58], and by metabolic remodeling [59]. Importantly, FUT4, FUT5, FUT6, and FUT7 have been confirmed as NF-κB targets [49,60,61].

CD28 expression is altered following DGH or TMZ treatment in glioma [11]. In our study, several CD28-associated pathways included GTN genes and involved PI3K/Akt signaling (co-stimulation by the CD28 family; CD28-dependent Vav1 pathway; CD28-dependent). These pathways might represent the significance of targeting the GTN gene network in glioma, which also involves the established PI3K/Akt activity in glioma [62,63]—an enriched pathway with GTN genes (WP4141). Significantly, CD28-mediated NF-κB activation [64,65] supports the notion that a positive feedback loop exists between CD28 and NF-κB, and supports the position of CD28 as a central molecule in the GTN network.

CD44 is a transmembrane glycoprotein that is expressed in most cell types, and is a receptor for hyaluronic acid and a marker of cancer stem cells, which is upregulated by NF-κB [50]. In glioblastoma, CD44 downregulation prevents tumor growth and sensitizes cancer cells to cytotoxic drugs [66]. The CD58 marker is a lymphocyte adhesion molecule, found to be overexpressed in gliomas associated with normal brain tissue [67]. This molecule is important for immune response, and CD58+ glioma cells are capable of causing tumor onset in vivo [68], while a significant decrease in CD58 expression by DGH suggests possible modified immunological responses in gliomas. CD71 corresponds to the transferrin-1 receptor (TfR1), and is associated with iron homeostasis at the cellular and organism level. CD71 expression is known to be elevated in various types of malignancy [69], and is an indicator of poor prognosis in various types of cancer, including glioblastoma [70]. Alteration of CD71 expression can also lead to disruption of intracellular iron levels, known to be associated with oxidative stress induction [71], which may represent a mechanism to sensitize glioma cells to cell death, or may inhibit glioblastoma cells from performing important biochemical functions. This conceptual framework is consistent with this study’s results, where GTN genes were associated with iron homeostasis (iron uptake and transport; transferrin endocytosis and recycling) and iron metabolism in the placenta (WP2007).

An interesting subsection of the suggested regulatory network involves the presence of IL2RB—a receptor for IL-2—as well as IL-12a. IL-12 is associated with a Th1-type anticancer immune response, and is capable of eliciting potent antitumor activity [72], as well as regulating the transcription of matrix metalloproteinases [73]. Cytokine signaling is tightly connected with NF-κB activity, and takes part in known networks that connect cancer with inflammation and immune response [14,74]. Cytokine signaling may also be associated with an orchestrated alteration of immune cell subtypes, cytokine levels, and an altered metabolite profile [74].

Collectively, our results support the presence of an expanded therapy-associated network of cluster of differentiation markers containing NF-κB cCREs, providing a comprehensive and unified mechanism of action for antiglioma therapy.

## Figures and Tables

**Table 1:** Most significant pathways containing GTN genes. The 25 most relevant pathways sorted by *p*-value are shown.

Pathway Name	Entities				Reactions	
	Found	Ratio	*p*-Value	FDR *	Found	Ratio
Lewis blood group biosynthesis	8/39	0.003	1.78 × 10^−13^	1.94 × 10^−11^	5/13	9.53 × 10^−4^
Blood group systems biosynthesis	8/52	0.003	1.73 × 10^−12^	9.37 × 10^−11^	5/22	0.002
Insulin-like Growth Factor-2 mRNA Binding Proteins (IGF2BPs/IMPs/VICKZs) bind RNA	5/13	8.63 × 10^−4^	3.49 × 10^−10^	1.26 × 10^−8^	2/3	2.20 × 10^−4^
Interleukin-10 signaling	6/86	0.006	1.31 × 10^−7^	3.53 × 10^−6^	2/15	0.001
Metabolism of carbohydrates	9/457	0.03	3.15 × 10^−6^	5.79 × 10^−5^	8/243	0.018
Iron uptake and transport	5/83	0.006	3.22 × 10^−6^	5.79 × 10^−5^	9/34	0.002
Transferrin endocytosis and recycling	4/39	0.003	4.21 × 10^−6^	6.31 × 10^−5^	7/11	8.06 × 10^−4^
Cell surface interactions at the vascular wall	7/257	0.017	5.67 × 10^−6^	7.37 × 10^−5^	4/65	0.005
CD28 dependent Vav1 pathway	3/17	0.001	1.48 × 10^−5^	1.78 × 10^−4^	5/6	4.40 × 10^−4^
CTLA4 inhibitory signaling	3/25	0.002	4.64 × 10^−5^	4.64 × 10^−4^	3/5	3.66 × 10^−4^
CD28 dependent PI3K/Akt signaling	3/26	0.002	5.21 × 10^−5^	4.69 × 10^−4^	2/9	6.60 × 10^−4^
Costimulation by the CD28 family	4/97	0.006	1.44 × 10^−4^	0.001	14/35	0.003
CD28 co-stimulation	3/39	0.003	1.72 × 10^−4^	0.001	11/19	0.001
Immune System	17/2684	0.178	3.46 × 10^−4^	0.002	97/1625	0.119
RUNX3 Regulates Immune Response and Cell Migration	2/10	6.64 × 10^−4^	3.55 × 10^−4^	0.002	2/5	3.66 × 10^−4^
Interleukin-35 Signalling	2/16	0.001	8.99 × 10^−4^	0.005	24/26	0.002
Hemostasis	8/803	0.053	0.001	0.008	5/334	0.024
Signaling by Interleukins	7/643	0.043	0.002	0.008	71/493	0.036
Integrin cell surface interactions	3/86	0.006	0.002	0.008	4/55	0.004
Cytokine Signaling in Immune system	9/1092	0.072	0.002	0.011	73/710	0.052
Constitutive Signaling by Aberrant PI3K in Cancer	3/96	0.006	0.002	0.011	1/2	1.47 × 10^−4^
PI3K/AKT Signaling in Cancer	3/124	0.008	0.005	0.019	1/21	0.002
Adaptive Immune System	8/1005	0.067	0.005	0.02	17/264	0.019
PI5P, PP2A and IER3 Regulate PI3K/AKT Signaling	3/129	0.009	0.005	0.021	1/7	5.13 × 10^−4^
Negative regulation of the PI3K/AKT network	3/137	0.009	0.006	0.025	1/10	7.33 × 10^−4^

* False Discovery Rate.

## Data Availability

Data is contained within article and Supplementary Materials.

## Supplementary Materials

The following are available online at https://www.mdpi.com/article/10.3390/biomedicines10050935/s1, Figure S1: Distance of NF-kB binding sites to human genes promoters, Table S1: NF-kB binding related to concesus cis-regulatory elements, Table S2: NF-κB binding sites in relation to human gene promoters.
