# Supplementary material for: An NF-κB- and Therapy-Related Regulatory Network in Glioma: A Potential Mechanism of Action for Natural Antiglioma Agents
Source: Biomedicines. 2022 Apr 19;10(5):935. doi: 10.3390/biomedicines10050935 (PMC9138293; doi:10.3390/biomedicines10050935)
Supplement: Supplementary file 1 [file biomedicines-10-00935-s001.zip › Figure S1.pdf]

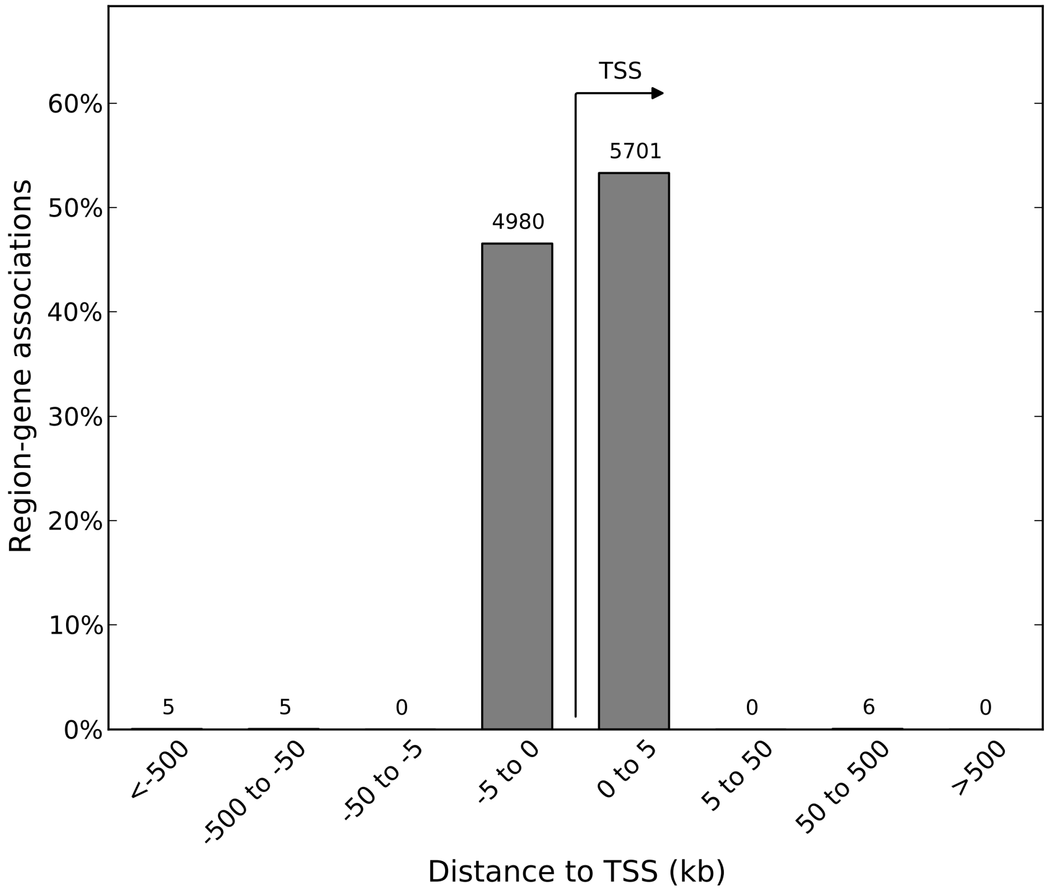

Figure S1: Distance of NF-kB binding sites to human genes promoters. The distance to Transcription Start Sites (TSS) was calculated implementing GREAT analysis.Distances to Transcription Start Sites (TSS) are shown, divided into 0-5, 5-50, 50-500 and >500 kb.
